# Lower Arginine Bioavailability, Increased FeNO Levels, and Airway Resistance on Impulse Oscillometry Are Characteristics of Asthma in Children and Young Adults with Sickle Cell Disease

**DOI:** 10.3390/medicina60030446

**Published:** 2024-03-08

**Authors:** Aylin Kont Ozhan, Tugba Arikoglu, Melih Er, Selma Unal, Didem Derici Yıldırım, Funda Erkasar, Şenay Balcı, Lulufer Tamer, Semanur Kuyucu

**Affiliations:** 1 Department of Pediatric Allergy and Immunology, Faculty of Medicine, Mersin University, Mersin 33110, Turkey; arikoglutugba@mersin.edu.tr (T.A.); semanurkuyucu@yahoo.com (S.K.); 2 Department of Pediatrics, Mersin City Training and Research Hospital, Mersin 33110, Turkey; meliher10@gmail.com; 3 Department of Pediatric Hematology, Faculty of Medicine, Mersin University, Mersin 33110, Turkey; unalselma@hotmail.com; 4 Department of Biostatistics, Faculty of Medicine, Mersin University, Mersin 33110, Turkey; didemderici@hotmail.com; 5 Department of Pediatric Hematology, Mersin City Training and Research Hospital, Mersin 33110, Turkey; fundaerci@yahoo.com; 6 Department of Biochemistry, Faculty of Medicine, Mersin University, Mersin 33110, Turkey; sbfidanci@hotmail.com (Ş.B.); lutamer@yahoo.com (L.T.)

**Keywords:** sickle cell disease, asthma, exhaled nitric oxide, arginine bioavailability, spirometry, impulse oscillometry, small airway dysfunction

## Abstract

*Background and Objectives*: Data on characteristics of asthma in children with sickle cell disease (SCD) is conflicting. Recently, the L-arginine pathway has gained attention in the pathogenesis of asthma and SCD. This study aimed to determine the distinctive clinical and laboratory features and the role of arginine metabolism in asthmatic children with SCD. *Materials and Methods*: A total of 52 children and adolescents with SCD, including 24 with asthma (SCD-A) and 28 without asthma (SCD-NA), and 40 healthy controls were included. A questionnaire, atopy tests, fractional exhaled nitric oxide (FeNO), and lung function tests were employed. Serum metabolites of the arginine pathway were measured. The results of the three groups were compared. *Results*: The demographic characteristics and atopy markers of the three groups were similar. FEV1%, FEV1/FVC, MMEF%, and total lung capacity (TLC%) values of SCD-A patients were not significantly different from the SCD-NA group, but they were significantly lower than the values measured in the controls. FeNO values greater than 35 ppb were present only in the SCD-A group. In impulse oscillometry, median resistance values at 5 Hz (R5)% were higher in both SCD subgroups than in healthy controls (*p* = 0.001). The (R5-20/R5)% values were higher in the SCD-A group (*p* = 0.028). Serum arginine levels and arginine bioavailability indices were significantly lower in the SCD-A group than in the SCD-NA group and healthy controls (*p* = 0.003 and *p* < 0.001). *Conclusions*: Asthma in children with SCD was not associated with atopy or low FEV1/FVC levels. However, lower arginine bioavailability and higher FeNO levels differentiated asthma in patients with SCD. High R5% and (R5-20/R5)% values indicated increased airway resistance in SCD, with a predominance of small airway disease in asthmatics.

## 1. Introduction

Asthma is a common chronic respiratory disease in patients with sickle cell disease (SCD) with a prevalence of 30–70% [1,2,3,4]. However, little is known about the pathophysiology underlying asthma in SCD. Given that asthma is often associated with acute chest syndrome (ACS) and the pathomechanisms involved are complex, making a diagnosis of asthma in children with SCD can be more challenging [5,6,7,8].

Abnormal lung function is commonly reported in patients with SCD. The obstructive pattern is the most commonly identified functional abnormality in young children, whereas the restrictive pattern may represent chronic disease progression from childhood to adulthood [9,10]. Many studies highlighted the importance of early lung function testing in the evaluation of children with SCD and respiratory symptoms [10,11]. In this sense, impulse oscillometry (IOS) is a noninvasive pulmonary function test that assesses respiratory mechanics during tidal breathing and can be employed as early as 2 to 3 years of age [12]. It has also been shown that the increase in IOS resistance values can precede detectable changes in spirometry in obstructive disease. Therefore, in patients unable to perform spirometry, such as toddlers, or in patients with normal spirometry, IOS can be used to determine the presence of increased airway resistance [13,14].

Although the lung is a well-known target of injury in SCD, there is limited evidence on airway inflammation in patients with SCD. While classic asthma is associated with a TH2 inflammatory pattern with elevated serum IgE levels and often positive allergy tests, inflammation in patients with SCD may be associated with different inflammatory pathways [15]. Fractional exhaled nitric oxide (FeNO) serves as a biomarker for eosinophilic airway inflammation in children with asthma. However, studies in children with SCD have produced conflicting reports [16,17,18]. In addition, some studies suggest that children with SCD may be predisposed to increased allergic inflammation [19,20].

Patients with SCD may potentially be at risk for chronic lung disease triggered by hemolysis-driven dysregulation of arginine NO metabolism [21]. A growing body of evidence suggests that dysregulation of arginine metabolism contributes to the pathogenesis of asthma through altered NO metabolism [22,23]. Therefore, indices of arginine metabolism could differentiate asthma phenotypes in children with SCD [24].

The literature highlights the need to evaluate the clinical and laboratory features of SCD patients with asthma. Thus, this study aimed to determine the clinical features, atopy markers, lung functions, and the role of L-arginine metabolism in asthmatic children and young adults with SCD to reveal the characteristics of asthma in SCD patients.

## 2. Materials and Methods

### 2.1. Study Population

The study population included patients with SCD and healthy controls. Children and young adults aged 7–24 years with SCD who were followed up with in the Pediatric Hematology Departments of Mersin province were included. Exclusion criteria for the cases were a history of upper and lower respiratory tract infections in the last month, painful crises, ACS or blood transfusion in the last 2 months, and inability to perform lung function tests. Healthy controls were age- and sex-matched children and young adults without SCD. Subjects with chronic respiratory, organ-specific, or systemic disease were excluded.

### 2.2. Study Design

Clinical data were collected by interviewing parents and reviewing medical records at enrollment. A comprehensive questionnaire was used to assess the demographic characteristics, allergy and respiratory symptoms, and medical history relevant to SCD. Medical charts of the children were reviewed to determine hydroxyurea use, transfusion history, ACS, and painful crises in the past year. ACS was defined as an episode of acute respiratory distress with a new pulmonary infiltrate on a chest radiograph, fever, and increased respiratory effort. Reactive airway symptoms were investigated by asking parents the following questions derived from the ISAAC questionnaire [25]: (1) Has your child had any wheezing or whistling on the chest in the last year? (2) Has your child had wheezing or whistling on the chest during or after physical exercise in the last year? (3) Has your child had wheezing or whistling on the chest without having flu in the last year? (4) Has your child had dyspnea or wheezing accompanied by dyspnea in the last year?

A skin prick test with inhalant allergens, blood sampling for atopy markers and arginine metabolites, and an FeNO test were applied on day one. Lung function tests were evaluated with IOS, spirometry, and plethysmography on the second day. IOS was performed before spirometry because of the effects of forced expiratory maneuvers on bronchial motor tone. The next day, bronchial hyperreactivity (BHR) was assessed by the methacholine bronchial challenge test. The study was approved by the Institutional Ethics Committee of Mersin University. All patients provided written informed consent before participating in the study.

### 2.3. Description of Study Cases

Children with SCD were defined as having asthma (SCD-A) if they had asthma-related reactive airway symptoms (cough, wheezing, dyspnea, and chest tightness) in the last year and demonstrated either airway reversibility (positive bronchodilator response in spirometry) or bronchial hyperreactivity in the methacholine provocation test [26,27]. SCD patients were defined as non-asthmatics (SCD-NA) if they had neither reactive airway symptoms nor BDR or BHR. SCD patients who did not meet these characteristics were not included in the study.

### 2.4. Atopy Markers

Atopic sensitization was determined by serum total IgE, inhalant-specific IgE, and a skin prick test. In addition to histamine and saline controls, the following inhalant antigens were applied: *Dermatophagoides pteronyssinus*, *Dermatophagoides farinae*, *Alternaria alternata*, cat and dog dander, mixed grass, ragweed, tree pollen, and cockroach. A positive skin prick test was defined as a mean diameter of the wheal at least 3 mm larger than the negative control, with surrounding erythema. Atopy was defined as specific IgE (>0.35 kU/L) or skin prick test positivity to any inhalant allergen. 

### 2.5. Measurement of Arginine Metabolites

Serum levels of L-arginine, asymmetric dimethylarginine (ADMA), citrulline, ornithine (Sunred Biotechnology, Shanghai, China), and spermine (Mybiosource, San Diego, CA, USA) were measured by an enzyme-linked immunosorbent assay. The bioavailability of arginine was determined by the ratio of arginine to the products generated from enzymatic degradation (arginine/ornithine + citrulline, arginine/spermine, and arginine/ADMA) in participants. 

### 2.6. Fractional Exhaled NO Measurement

FeNO measurement was performed with the NIOX system (Aerocrine AB, Solna Sweden) according to American Thoracic Society (ATS) guidelines before spirometry, because forced expiration can lower FeNO values [28]. Participants were instructed to exhale to residual volume; after insertion of the mouthpiece, they were asked to inhale to total lung capacity. The patient then exhaled at a constant flow rate of 50 mL/s for 10 s. FeNO levels above 35 ppb in children with asthma-related symptoms indicate significant eosinophilic inflammation and support a diagnosis of asthma according to the ATS guideline [28]. 

### 2.7. Pulmonary Function Tests

#### 2.7.1. Spirometry

Spirometry was performed by a Master Screen Spirometry System (JaegerCo, Hoechberg, Germany) according to the ATS guideline [29]. The values of forced expiratory volume in 1 s (FEV1), forced vital capacity (FVC), FEV1/FVC ratio, and maximum mid-expiratory flow (MMEF) were recorded. Spirometric indices were expressed as percent of the predicted values. Bronchodilator response was calculated after inhalation of 200–400 mcg salbutamol. A positive bronchodilator response (BDR) is defined as an increase in FEV1 of at least 12% and 200 mL from baseline in young adults and an increase of at least 12% or 200 mL from baseline in children after inhalation of salbutamol, as defined by the Global Initiative for Asthma (GINA) guideline [26]. Because salbutamol inhalation was not considered ethical in healthy children, BDR was not assessed in healthy controls.

#### 2.7.2. Impulse Oscillometry

Impulse oscillometry (IOS) was performed in accordance with the European Respiratory Society/American Thoracic Society guidelines (ERS/ATS) [30]. The MasterScreen IOS system (JaegerCo, Hoechberg, Germany) was used. The output pressure and flow signals were analyzed for 30 s in the frequency range of 5–20 Hz for their amplitude and phase differences to determine the resistance. The IOS parameters obtained at the end of the application were the resistances at 5 and 20 Hz (R5, R20) and (R5-20)%. (R5-20)% was calculated accordingly: (R5-20)/R5 × 100. IOS was performed before FeNO measurement and lung function tests. 

#### 2.7.3. Plethysmography

Total lung capacity (TLC) was determined using a MasterScope Body plethysmography system (JaegerCo, Hoechberg, Germany) according to the ATS guideline, and the results were reported as percent predicted for age, sex, and height [31].

#### 2.7.4. Methacholine Bronchial Provocation Test

The methacholine challenge test was performed according to the ATS guideline [32]. The test was not applied to patients with current wheeze or dyspnea or a baseline SaO2 of <92% or FEV1 < 70%. The bronchial challenge test was performed with a 2 min tidal breathing method. Bronchial hyperreactivity (BHR) was defined as a positive methacholine challenge test with a PC20 value < 16 mg/mL according to the ATS guideline [32]. Responsiveness to methacholine was also determined by dose–response slope (DRS). In calculating the DRS, the numerator of the slope was the percent decrease in FEV1 from the post-saline value to the last concentration of methacholine used in testing, and the denominator was the cumulative methacholine dose. 

### 2.8. Statistical Analysis

Statistica version 13.5.0.17 software was used for statistical analyses. Descriptive analyses were performed using median values (25–75 percentile) or means ± standard deviations (SDs), depending on normality demonstrated by the Kolmogorov–Smirnov test. The study groups were compared using the one-way ANOVA and the Kruskal–Wallis tests, and a Bonferroni correction was applied as a post hoc test. The Mann–Whitney U test was used to compare two groups when the variable was continuous, but not normally distributed. Categorical endpoints were summarized as percentages, and significant differences or associations were analyzed with the X^2^ test or Fisher’s exact test. A logarithmic transformation was performed to deal with skewed data. A *p*-value of less than 0.05 was considered statistically significant.

## 3. Results

### 3.1. Patient Characteristics and Demographics

A total of 52 children and young adults with SCD (24 with SCD-A and 28 with SCD-NA) and 40 healthy controls were included in the study. The median age was 15.0 years in the SCD groups and healthy controls. There was no significant difference between groups in terms of age, gender, and BMI (*p* = 0.937, *p* = 0.273, and *p* = 0.344, respectively). No significant difference was found between SCD-A and SCD-NA subgroups concerning the SCD genotype, history of ACS and painful crises, and hydroxyurea use. The hemoglobin levels of SCD patients were lower than those of the healthy controls (*p* < 0.001) (Table 1).

There was no statistically significant difference between all groups in terms of serum total IgE level, eosinophil count, personal atopy, and family history of atopy. The number of patients with FeNO levels greater than 35 ppb was significantly higher in the SCD-A group (*p* = 0.001). The clinical and demographic characteristics of patients with SCD and healthy controls are shown in Table 1. No correlation was found between FeNO levels and atopy and clinical characteristics of SCD.

### 3.2. Comparison of Lung Function Tests of SCD Patients and Healthy Controls

In spirometry, FEV1% and MMEF% pred values of the SCD-A group were significantly lower than those of the SCD-NA group and healthy controls (*p* = <0.001, *p* = 0.025). ΔFEV1% and ΔMMEF% values (bronchodilator reversibility in FEV1 and MMEF) of the SCD-A group were significantly higher than those of SCD-NA group (*p* = 0.021, *p* = <0.001). No significant difference was found between the groups regarding FEV1/FVC values. In IOS, R5% pred values of both SCD subgroups were significantly higher than healthy controls (*p* = 0.001). ΔR5% values (bronchodilator reversibility in R5) of the SCD-A group were significantly higher than the SCD-NA group (*p* = 0.003). The frequency-dependent resistance values (R5-20/R5)% of the SCD-A group were significantly different from those of the SCD-NA group, but higher than healthy controls (*p* = 0.028). In plethysmography, TLC% pred levels of the SCD-A group were significantly lower than those of healthy controls (*p* = 0.006), but not than the SCD-NA group. DRS methacholine values were significantly higher in the SCD-A group than in the SCD-NA group in the bronchial provocation test (Table 2).

### 3.3. Comparison of Serum Arginine and Bioavailability Indices of SCD Patients and Healthy Controls

Serum arginine levels were significantly lower in the SCD-A group than the SCD-NA group and healthy controls (*p* = 0.003). Arginine bioavailability indices, determined by arginine/ornitine + citrulline, arginine/spermine, and arginine/ADMA levels, were significantly lower in the SCD-A group than the SCD-NA group and healthy controls (*p* = <0.001, *p* = 0.005, and *p* = <0.001, respectively) (Table 3).

## 4. Discussion

Asthma is a complex chronic respiratory disease characterized by chronic airway inflammation, bronchial hyperreactivity, and remodeling [26]. Asthma is common in children with SCD and appears to be associated with increased morbidity [3,33]. Despite the high burden of asthma, there is a paucity of data on the pathogenesis, clinical, and lung function characteristics of asthma associated with SCD.

The pathomechanism underlying asthma in SCD patients may differ from classic allergic asthma. While classic allergic asthma in the general population is associated with T-helper 2 inflammation with increased serum IgE levels and often positive allergy tests, the inflammation in SCD patients involves different inflammatory pathways [15]. Besides that, the results of studies investigating whether IgE levels can be used to distinguish individuals with and without asthma in SCD are inconsistent [15,34]. In the present study, no significant difference was found regarding serum eosinophil count, total IgE levels, personal atopy, and family history of atopy in SCD-A and SCD-NA patients compared to healthy controls. In agreement with our results, Strunk et al. showed that there was no difference in IgE levels between children with SCD with and without asthma. However, they showed that having ≥2 positive skin tests for aeroallergens was more frequent among SCD patients with asthma compared to those without. Parental history of asthma also proved to be important in distinguishing children with SCD with a physician diagnosis of asthma [34]. In the present study, neither atopic characteristics nor family history were able to discriminate between SCD-A and SCD-NA patients. A clinical diagnosis of asthma has also been associated with increased rates of ACS and painful crises in SCD patients [35]. However, we found no significant difference between the SCD-A and SCD-NA groups with respect to a history of ACS or painful crises, hydroxyurea use, and genotype.

It has been the topic of debate whether the high prevalence of asthma in SCD patients is due to the contribution of overlapping mechanisms shared between these otherwise distinct disorders. In that sense, an increasing number of studies have shown that arginine metabolism is impaired in both SCD and asthma, suggesting that arginine deficiency may be a common crossroad in the pathophysiology [1,6,36]. In the scope of asthma and SCD comorbidity, heightened baseline inflammation may lead to increased expression of both inducible NO synthase (NOS) and arginase, thereby further depleting cellular arginine pools. L-arginine is a common substrate for arginase for the biosynthesis of ornithine, which can serve as a precursor for the synthesis of proline and polyamines (spermine), and thereby contributes to collagen deposition and cell proliferation, leading to airway remodeling and hyperreactivity. In addition to arginase, the enzyme NOS uses L-arginine for the generation of NO and citrulline [6,15,37]. The current study extends our understanding of the arginine–NO metabolic pathway in asthma and SCD, as it is the first study to investigate L-arginine metabolism among SCD patients with and without asthma. We demonstrated that serum arginine levels and arginine bioavailability indices (arginine/spermine and arginine/ornithine and citrulline) were significantly lower in the SCD-A group compared to other groups. An imbalance of L-arginine and ADMA, an inhibitor of the constitutive forms of NOS, causes a greater oxidative stress in the airways by uncoupling NOS, leading to the formation of superoxide over NO [38,39]. Consistent with these data, we found that the arginine/ADMA ratio, a marker of NOS functional impairment, was significantly lower in the SCD-A group. However, despite the lower arginine levels, FeNO levels above 35 ppb were present only in the SCD-A group. This paradoxical increase in FeNO can be explained in two ways: First, serum arginine levels may not reflect the levels of intracellular arginine in the airways [40]. Second, production of NO from nitrites may occur in a non-enzymatic manner independent of arginine bioavailability due to airway acidification in patients with asthma [41].

Much attention has been directed towards measurement of FeNO in asthma, as it is considered a noninvasive biomarker of airway inflammation [42]. FeNO has been associated with several features of atopy and asthma, including serum total IgE levels, peripheral blood eosinophilia, FEV1/FVC ratio, and airway hyperreactivity in school-aged children and adolescents [43,44]. However, the value of FeNO measurement in SCD is not well known. Previous studies have shown that FeNO levels in children with SCD are higher, lower, or the same as in healthy controls [17,18,45]. Furthermore, a recent study found a significant positive correlation between pulmonary blood flow and alveolar NO in the SCD group, indicating that the production of NO by the alveolar compartment of the lung is associated with chronic hyperdynamic circulation in patients with SCD. They suggested that the FeNO of a patient with SCD is to some extent due to increased alveolar NO production as a result of chronic anemia rather than airway inflammation due to asthma [46]. In the present study, patients with higher FeNO levels were much more common in the SCD-A group compared to SCD-NA and healthy groups. However, we found no correlation between FeNO levels and atopy features in SCD, suggesting alternative forms of airway inflammation in SCD that differ from the classic pathways associated with childhood atopic asthma. 

It is necessary to monitor lung function in SCD patients from early childhood since lower airway obstruction has been reported to be common in children with SCD and has been described as early as in infancy [2,4,47]. Although the importance of lower airway obstruction in patients with SCD is not well understood, it is known to be associated with increased rates of hospitalization for ACS or painful crises [48]. Koumbourlis et al. showed that lower airway obstruction was the most common abnormality of lung function in children with SCD. While obstructive airway disease is common in younger children with SCD, the restrictive pattern gradually becomes predominant as a sequela of recurrent ACS and chronic inflammation [9,10]. A decrease in lung volume indices, especially TLC, helps to diagnose a restrictive pattern in plethysmography [49]. In the present study, despite symptomatic airway reversibility, FEV1% and TLC% values were significantly lower in SCD-A patients compared to healthy controls but did not differ from SCD-NA patients. However, the FEV1/FVC ratio of the SCD-A group did not differ from that of SCD-NA patients and healthy controls. These results pointed to a decrease in both FEV1 and FVC values leading to a normal FEV1/FVC ratio in all children with SCD, regardless of an asthma diagnosis. Since our cohort included adolescents and young adults, a restrictive or mixed disease process seemed to be predominant. Therefore, the FEV1/FVC ratio, which is the most sensitive parameter of obstructive lung disease, may not be useful for the diagnosis of asthma in SCD patients, especially in older children and adolescents. 

Among younger patients with SCD, reversible lower airway obstruction primarily affects the smaller airways and precedes the development of restrictive lung disease [2]. In the current study, although MMEF% values were within the normal range, bronchodilator responsiveness in MMEF was significantly higher in the SCD-A group compared to the SCD-NA group, pointing to the importance of reversibility in expiratory flow rates in the diagnosis of asthma.

IOS provides valuable insight into respiratory mechanics. It requires only passive cooperation and can be easily applied even to young children [12,50]. IOS was used in the present study to assess respiratory system resistance, as this technique is more sensitive than spirometry in assessing peripheral airway function [13,14,51]. There is very little data on IOS findings in SCD patients with respiratory symptoms [50]. These studies suggest that worsening of airway resistance in SCD children may be related to increased pulmonary capillary blood volume in addition to asthma [46,52]. In the present study, all SCD patients, whether or not they had asthma, had higher respiratory system resistance (R5%) compared to healthy controls. The raised R5% values in SCD children, both with and without asthma, could be due to the increased pulmonary capillary blood volume as a result of elevated cardiac output due to chronic anemia [46,52]. However, we were unable to measure pulmonary capillary blood volume to support these data. R5-20 reflects the elastic properties of the peripheral lung and thus provides information about changes in the caliber of peripheral airways [53,54]. In the present study, the (R5-20/R5)% values of SCD-A children were significantly higher than in the healthy group, which may indicate small airway dysfunction in SCD patients with asthma despite the normal MMEF values in spirometry.

A limitation of our study was the small sample size, which may have limited our ability to detect some other differences between SCD patients with and without asthma. However, although the number of SCD cases in the study subgroups was low, there is no statistically significant discrepancy between the patient subgroups and healthy controls in terms of the number of patients that would grossly affect the results. In addition, we were unable to measure pulmonary capillary blood volume in all SCD patients to clarify the etiology of increased total respiratory resistance. The strengths of the present study were the objective assessment of SCD patients with a wide range of simultaneous lung function tests, including spirometry, IOS, plethysmography, and methacholine challenge testing. In addition, the present study provided evidence that the characteristic features of asthma in SCD are not similar to classic allergic asthma. To the best of our knowledge, this is the first study comparing arginine–NO metabolism in children with SCD and young adults with and without asthma. Given the limited data on the pathophysiology of asthma in SCD, our study provided additional insight into the complex mechanisms underlying asthma. Notably, data came from two centers, so it may not be generalized to the general population. 

In conclusion, asthma in children with SCD might not be associated with atopy, eosinophilia, or low FEV1/FVC values, and can have some features divergent from classic allergic asthma. It involves a combination of several mechanisms. In IOS, the increased total respiratory resistance in all SCD patients, and particularly the frequency-dependent resistance values in asthmatics, suggests that pathophysiologic factors specific to SCD itself may result in resistive airway disease. This may not always present as a clinical asthma phenotype, but may sometimes be a precursor of silent or overwhelming airway disease. Our study also showed that dysregulation of the arginine–NO pathway, a common crossroad in the pathophysiology of both SCD and asthma, was one of the characteristics of asthma patients with SCD. A better understanding of the complex mechanisms underlying airway inflammation and respiratory mechanics in SCD would allow a more appropriate endophenotypic definition of asthma in this particular group. Furthermore, in the face of such divergent features of asthma in SCD patients, the optimal treatment is still unclear and requires further controlled studies.

## Figures and Tables

**Table 1:** The comparison of clinical characteristics and atopy markers in subgroups of sickle cell disease patients and healthy controls.

Variable	SCD with Asthma (SCD-A) n = 24	SCD without Asthma (SCD-NA) n = 28	Healthy Controls n = 40	*p*-Value
Age in years	15.0 (12.0–17.0)	15.0 (12.0–18.7)	15.0 (11.0–20.2)	0.937
Male sex n (%)	15.0 (62.5)	20.0 (71.4)	21.0 (52.5)	0.273
BMI (kg/m^2^)	18.1 (17.3–21.9)	18.1 (16.2–22.5)	20.1 (17.5–23.4)	0.344
Hb (g/dL)	9.2 (8.1–9.9) ^a^	9.0 (8.2–10.2) ^b^	13.1 (12.5–14.1)	**<0.001**
Serum absolute eosinophil count (cells/mm^3^)	187.5 (93.5–357.7)	154.5 (95.5–349.7)	134.5 (70.0–268.3)	0.389
Serum total IgE (IU/mL)	71.0 (28.0–130.0)	54.5 (21.5–113.2)	41.5 (22.0–70.0)	0.262
FeNO (ppb)				
<20 ppb n (%)	18 (75.0)	20 (71.4)	37 (92.5)	0.056
>35 ppb n (%)	4 (23.5) ^a,c^	0 (0)	0 (0)	**0.001**
Subjects with atopy * n (%)	2.0 (8.3)	3.0 (10.7)	5.0 (12.5)	0.916
A family history of atopy/asthma n (%)	6.0 (25.0)	2.0 (7.1)	2.0 (5.0)	0.059
A history of ACS in the last year n (%)	5.0 (20.8)	7.0 (25.0)	-	0.722
A history of painful crisis in the last year n (%)	18.0 (75.0)	19.0 (67.9)	-	0.571
Current hydroxyurea use n (%)	17.0 (70.8)	21.0 (75.0)	-	0.736
HbSS Genotype n (%)	21.0 (87.5)	21.0 (75.0)	-	0.309

Results in median (25–75 percentile) or mean ± SD. * Atopy was defined as specific IgE positivity or skin prick test positivity to any inhalant allergen. ACS: Acute chest syndrome, BMI: Body mass index, Hb: Hemoglobin, FeNO: Fractional exhaled nitric oxide. SCD with asthma (SCD-A): Sickle cell disease patients with reactive airway symptoms and BDR or BHR. SCD without asthma (SCD-NA): Sickle cell disease patients with neither airway symptoms nor BDR or BHR. ^a^ Significant difference between SCD-A patients and healthy controls. ^b^ Significant difference between SCD-NA patients and healthy controls. ^c^ Significant difference between SCD-A patients and SCD-NA patients. The results found as statistically significant are shown in bold.

**Table 2:** The comparison of pulmonary function tests of subgroups of sickle cell disease patients and healthy controls.

Variable	SCD with Asthma (SCD-A) n = 24	SCD without Asthma (SCD-NA) n = 28	Healthy Controls n = 40	*p*-Value
**Spirometry**				
FEV1% predicted	94.2 ± 16.1 ^a^	102.1 ± 11.9	109.6 ± 15.3	**<0.001**
∆ FEV1%	5.7 (1.5–8.1)	2.5 (0.5–4.3)	–	**0.021**
FEV1/FVC	85.4 ± 5.6	86.7 ± 4.0	87.7 ± 7.6	0.868
MMEF% predicted	85.2 ± 21.2 ^a^	95.9 ± 16.5	103.0 ± 30.8	**0.025**
∆ MMEF%	22.8 (10.6–32.2)	7.8 (3.2–15.8)	–	**<0.001**
**Impulse oscillometry**				
R5% predicted	133.0 (108.3–160.6) ^a^	131.9 (111.6–154.6) ^b^	105.9 (89.6–132.9)	**0.001**
∆R5%	−20.5 (−28.4–−14.5)	−10.3 (−18.1–−6.2)	–	**0.003**
(R5-20/R5)%	30.9 ± 16.8 ^a^	22.9 ± 13.9	20.4 ± 15.1	**0.028**
**Plethysmography**				
TLC% predicted	91.9 ± 16.8 ^a^	95.0 ± 12.8	103.0 ± 12.6	**0.006**
RV/TLC %	91.4 (80.3–103.2)	80.6 (66.9–91.5)	90.6 (75.3–98.9)	0.108
**Bronchial provocation test**				
DRS methacholine	1.7 (0.4–3.7)	0.1 (−0.1–0.3)	–	**<0.001**

Results in median (25–75 percentile) or mean ± SD. ∆ FEV1%: % change in prebronchodilator FEV1 value after salbutamol. ∆MMEF %: % change in prebronchodilator MMEF value after salbutamol. ∆R5%: % change in prebronchodilator R5 value after salbutamol. DRS methacholine: Dose–response slope of methacholine provocation test. SCD with asthma (SCD-A): SCD patients with reactive airway symptoms and BDR or BHR. SCD without asthma (SCD-NA): SCD patients with neither airway symptoms nor BDR or BHR. ^a^ Significant difference between SCD patients with asthma and healthy controls. ^b^ Significant difference between SCD patients without asthma and healthy controls. The results found as statistically significant are shown in bold.

**Table 3:** The comparison of arginine metabolites in subgroups of SCD patients and healthy controls.

Variable	SCD with Asthma (SCD-A) n = 24	SCD without Asthma (SCD-NA) n = 28	Healthy Controls n = 40	*p*-Value
Log arginine	2.2 (1.4–2.9) ^a,b^	3.4 (1.9–3.5)	3.0 (2.6–3.6)	**0.003**
Log arginine/spermine	2.2 (0.8–3.1) ^a,b^	3.5 (1.9–3.7)	3.1 (2.6–3.8)	**0.005**
Log arginine/ornitine + citrulline	1.5 (1.0–2.1) ^a,b^	2.4 (1.8–2.5)	2.4 (2.3–2.5)	**<0.001**
Log arginine/ADMA	2.1 (1.4–2.6) ^a,b^	2.9 (1.8–3.1)	3.1 (2.8–3.2)	**<0.001**

^a^ Significance between SCD patients with asthma and healthy controls. ^b^ Significance between SCD patients with and without asthma. The results found as statistically significant are shown in bold.

## Data Availability

The data presented in this study are available upon request from the corresponding author.
